# The Mitochondria-Targeted Antioxidant SkQ1 Downregulates Aryl Hydrocarbon Receptor-Dependent Genes in the Retina of OXYS Rats with AMD-Like Retinopathy

**DOI:** 10.1155/2014/530943

**Published:** 2014-07-14

**Authors:** M. L. Perepechaeva, A. Yu. Grishanova, E. A. Rudnitskaya, N. G. Kolosova

**Affiliations:** ^1^Institute of Molecular Biology and Biophysics of Siberian Branch of RAMS, Timakova Street 2, Novosibirsk 630117, Russia; ^2^Institute of Cytology and Genetics, Prospekt Acad, Lavrentjeva 10, Novosibirsk 630090, Russia; ^3^Novosibirsk State University, Pirogova 2, Novosibirsk 630090, Russia

## Abstract

The mitochondria-targeted antioxidant SkQ1 is a novel drug thought to retard development of age-related diseases. It has been shown that SkQ1 reduces clinical signs of retinopathy in senescence-accelerated OXYS rats, which are a known animal model of human age-related macular degeneration (AMD). The aim of this work was to test whether SkQ1 affects transcriptional activity of *AhR* (aryl hydrocarbon receptor) and *Nrf2* (nuclear factor erythroid 2-related factor 2), which are considered as AMD-associated genes in the retina of OXYS and Wistar rats. Our results showed that only *AhR* and *AhR*-dependent genes were sensitive to SkQ1. Dietary supplementation with SkQ1 decreased the *AhR* mRNA level in both OXYS and Wistar rats. At baseline, the retinal *Cyp1a1* mRNA level was lower in OXYS rats. SkQ1 supplementation decreased the *Cyp1a1* mRNA level in Wistar rats, but this level remained unchanged in OXYS rats. Baseline *Cyp1a2* and *Cyp1b1* mRNA expression was stronger in OXYS than in Wistar rats. In the OXYS strain, *Cyp1a2* and *Cyp1b1* mRNA levels decreased as a result of SkQ1 supplementation. These data suggest that the Cyp1a2 and Cyp1b1 enzymes are involved in the pathogenesis of AMD-like retinopathy of OXYS rats and are possible therapeutic targets of SkQ1.

## 1. Introduction

Mitochondria-targeted antioxidant SkQ1 (cationic plastoquinone derivative (10-[6′-plastoquinonyl] decyltriphenylphosphonium) is a novel medication that is designed to retard the development of age-related diseases and aging [1, 2]. Plastoquinone, a very effective electron carrier and an antioxidant of chloroplasts, was conjugated with decyltriphenylphosphonium to obtain a cation that easily penetrates the cell membrane [3–5]. SkQ1's geroprotective properties are based on the ability of this reagent to attenuate pathological processes associated with production of reactive oxygen species [4]. The effects of SkQ1 on aging are accompanied by inhibition of development of such age-related problems as cataract, retinopathy, glaucoma, balding, canities, osteoporosis, involution of the thymus, and peroxidation of lipids and proteins [1, 3]. Recently it was shown that addition of SkQ1 to food or treatment with SkQ1 eye drops not only prevents development of retinopathy but also reduces severity of preexisting pathological changes in the retina of senescence-accelerated OXYS rats [6].

OXYS rats are a known animal model of human age-related macular degeneration (AMD) [7, 8]. OXYS rats develop a retinopathy similar to the dry form of human AMD judging by the symptoms, morphology, and some molecular changes. The OXYS retinopathy involves hypoplasia and atrophy of the retinal pigment epithelium and of photoreceptors, formation of drusen, and retinal neovascularization; this retinopathy also correlates with expression of VEGF (vascular endothelial growth factor) [8]. It was reported that the effects of SkQ1 include improvement of functions of the retinal pigment epithelium and a reduction of lipofuscin accumulation in the OXYS retina [9, 10]. The molecular mechanisms underlying the effects of SkQ1 have yet to be investigated.

AMD etiology includes genetic predisposition, exposure to environmental toxins and free radicals, and low levels of antioxidants. The pathogenesis of AMD is being actively studied but is not fully understood at present; several key factors are known to be involved. Oxidative stress is one of these key factors together with structural and functional changes in the retinal pigment epithelium, inflammation, and activation of the complement cascade. Recently, some genes that take part in the regulation of redox processes were characterized as possible AMD-associated genes. These genes include nuclear factor erythroid 2-related factor 2 (Nrf2) [11] and aryl hydrocarbon receptor (AhR) [12]. After analysis of haplotypes of detoxification genes,* AhR* caught the attention of researchers as a possible risk factor of AMD [12]. Studies of* AhR* ^−/−^ mice further support the potential role of this gene in AMD pathogenesis [13].

AhR is a ligand-dependent transcription factor that recognizes and binds to a wide range of xenobiotics and endogenous compounds. When inactive, AhR is located in the cytoplasm along with its associated proteins. After AhR binds to its ligand, the above-mentioned complex disintegrates, ligand-bound AhR moves to the nucleus, and dimerizes with Arnt (aryl hydrocarbon receptor nuclear translocator). The AhR/Arnt heterodimer then binds to xenobiotic-responsive elements (XREs) in the genomic DNA, and this process causes initiation of transcription of AhR's target genes [14, 15].

AhR controls expression of some components of phase I and phase II of xenobiotic metabolism [14, 15]. The following are phase I enzymes: cytochrome P450 1 subfamily (Cyp1a1, Cyp1a2, and Cyp1b1) and aldehyde dehydrogenase 3A1 (Aldh3a1) as a participant of phase II of the xenobiotic metabolism [16].

Nrf2 is a transcription factor that controls gene expression of antioxidant systems of the cell. In particular, it controls expression of heme oxygenase 1 (Hmox1), thioredoxin reductase 1 (Txnrd1), and glutathione S-reductase (Gsr) [17]. Nrf2, while in a complex with Maf proteins, interacts with antioxidant-responsive elements (ARE) in the promoter region of target genes [18].

Recent findings demonstrate a relationship between AhR-dependent and Nrf2-dependent signal transduction pathways; Nrf2 may be a genomic target of AhR, and the possibility of a cross-talk between the AhR/XRE and Nrf2/ARE signal transduction pathways cannot be ruled out [16, 19]. Some genes are controlled by both AhR and Nrf2. The list includes NAD(P)H: quinone oxidoreductase 1 (*Nqo1*), uracil diphosphate- (UDP-) glucuronosyltransferase 1A6 (*Ugt1a6*), UDP-glucuronosyltransferase (UGT) 1A9 (*Ugt1a9*), glutathione S-transferase (GST) A1 (*Gsta1*), and a number of other isoforms of UGT and GST [16]. Genes that are controlled by AhR, Nrf2, and both AhR and Nrf2 are sometimes called the* AhR-Nrf2 gene battery*.

The aim of this study was to test whether the antioxidant SkQ1 affects transcriptional activity of a redox-sensitive system: the AhR-Nrf2 gene battery. OXYS and Wistar rats received SkQ1 with food between the ages of 1.5 to 3 months, which is the period of active manifestation of signs of retinopathy. It was repeatedly proven previously that SkQ1 in this regimen can prevent the development of retinopathy in OXYS rats [6, 20].

## 2. Methods

### 2.1. Reagents

TRI-Reagent and RNA Secure Reagent were purchased from Ambion (USA); the cDNA synthesis MMLV RT kit and PCR kit qPCRmix-HS were from Evrogen (Russia); RNasin and RQ1 DNase were from Promega (USA); oligonucleotides (primers) for analysis of the rat genes* Cyp1a1*,* Cyp1a2*,* Cyp1b1*,* Gsta1*,* Nqo1*,* Aldh3a1*,* Ugt1a6*,* Ugt1a9*,* AhR*,* Nrf2*,* Gsr*,* Txnrd1*,* Hmox1*, and* Gapdh *were from Syntol (Russia). SkQ1 was synthesized as described earlier [5]. All other chemicals were obtained from other commercial sources and were analytical grade.

### 2.2. Animals

Male senescence-accelerated OXYS and age-matched male Wistar (control) rats were obtained from the Shared Center for Genetic Resources of Laboratory Animals of the Institute of Cytology and Genetics, Siberian Branch of the Russian Academy of Sciences (SB RAS; Novosibirsk, Russia). The rats were kept under standard laboratory conditions (at 22 ± 2°C, 60% relative humidity, and natural light), provided with standard rodent feed, PK-120-1, Ltd. (Laboratorsnab, Russia), and given water* ad libitum*. All experiments in this study were approved by the Institutional Review Board and performed in accordance with the Animal Care Regulations of the Institute of Cytology and Genetics (Novosibirsk) and with the international norms for studies on laboratory animals.

To assess the effects of SkQ1 (from the age of 1.5 months to the age of 3 months) on gene expression, 1.5-month-old male OXYS rats were randomly assigned to 1 of the 2 groups: the standard (control) diet or the diet supplemented with 250 nmol SkQ1 per kilogram of body weight per day (15 rats per group). The age-matched Wistar rats (standard diet) served as a control (15 rats in this group). The rats were euthanized using CO_2_ inhalation and killed by decapitation 5 days after the last examination of eyes. The retinas were removed, frozen, and stored at −80°C until analysis.

### 2.3. RNA Isolation and Reverse Transcription

Total RNA was isolated using the TRI-Reagent isolation kit (Ambion) as per the manufacturer's protocol. The RNA pellets were dissolved in 1 mM sodium citrate buffer pH 6.5, containing 1× RNA Secure Reagent (Ambion). The RNA concentration was measured using UV spectrophotometry. The RNA samples were treated with RNase-free DNase (Promega, USA) according to the manufacturer's instructions. Then the samples were subjected to repeated RNA extraction with a phenol-chloroform mixture and pure chloroform followed by precipitation with propanol. Reverse transcription was performed using the cDNA synthesis MMLV RT kit (Evrogen, Russia) according to the manufacturer's protocol.

### 2.4. Real-Time PCR

The PCR primer sequences used are presented in Table 1.

*Gapdh* served as an internal control (housekeeping gene). The gene expression patterns were analyzed using the iCycler CFX96 real-time PCR detection system (Bio-Rad Laboratories, USA) based on the TaqMan principle. Aliquots from all cDNA samples were mixed, and the “average” solution was used for preparation of calibration curves, which were used for measurement of relative cDNA levels of genes under study and of a reference gene in experimental samples. The reaction mixture contained the qPCRmix-HS buffer (Evrogen, Russia); a primer mix consisting of 0.5 *μ*L of 5 *μ*M probe, 1 *μ*L of a 10 *μ*M forward primer, and 1 *μ*L of a 10 *μ*M reverse primer; and 2000 ng cDNA. The reaction was conducted under the following conditions: heating at 95°C for 3 min, then 40 cycles of denaturation at 95°C for 15 s, and annealing/extension at 60°C for 30 s.

In each experiment, we added samples of cDNA under study with primers specific to a target gene (in triplicate for each cDNA sample) to wells of 1 multiwell plate, and similar samples with primers specific to a comparison gene were added to other wells of the same multiwell plate (also in triplicate). From these cDNA samples, we took identical amounts of cDNA to build a standard curve (this was an absolute quantification method using a standard curve). We used serial dilutions of the standard cDNA from 1 : 3 to 1 : 27. To wells of 1 multiwell plate, we added 2-3 repeats of reactions containing primers specific to a target gene and similar samples with primers specific to a comparison gene (2-3 repeats). Using the resulting standard curves, we quantified the original amount of cDNA (relative to the standard cDNA), and this value was normalized to the amount of cDNA of the comparison gene (*Gapdh*) [21]. For each cDNA sample, PCR was repeated at least twice.

### 2.5. Statistical Analysis

All calculations were performed using the* STATISTICA* software package (StatSoft, Inc., USA). All data were analyzed using two-way ANOVA and the Newman-Keuls* post hoc* test. The independent variables were genotype (Wistar, OXYS) and treatment (controls, SkQ1). One-way ANOVA was used for individual group comparison. The data are presented as mean ± SEM. The results were considered statistically significant if the *P* value was less than 0.05.

## 3. Results

In this work, we analyzed mRNA expression of AhR and AhR-dependent genes, Nrf2 and Nrf2-dependent genes, and AhR+Nrf2-dependent genes in phases I and II of xenobiotic metabolism.

**Figure 1:**
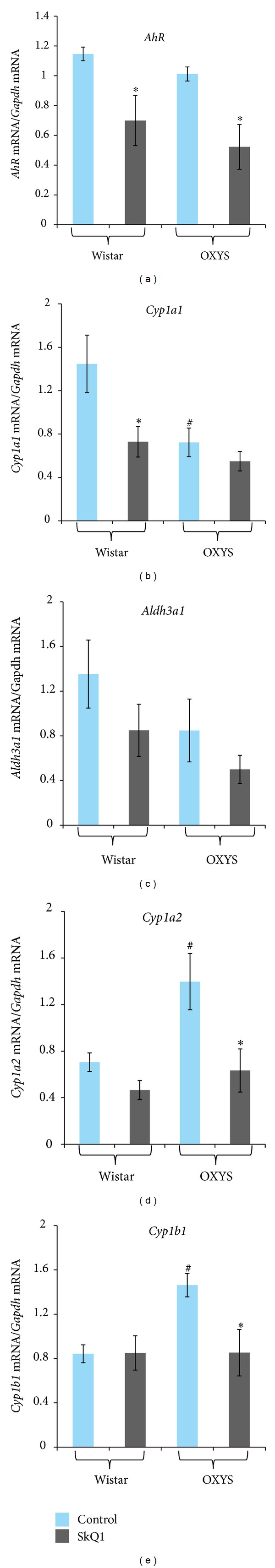
*AhR* (a),* Cyp1a1* (b),* Aldh3a1* (c),* Cyp1a2* (d), and* Cyp1b1* (e) mRNA levels in the retinas of 3-month-old Wistar and OXYS rats treated with SkQ1. All data are normalized to the expression level of a housekeeping gene (*Gapdh*). Values are presented as mean ± SEM (*n* = 6 to 8). Significant differences between groups are marked with *P < 0.05.

Figure 1 shows mRNA levels of AhR and AhR-dependent genes of phase I (*Cyp1a1*,* Cyp1a2*, and* Cyp1b1*) and phase II (*Aldh3a1*). Our data show that the genotype had no influence on the* AhR* mRNA level (Figure 1(a)) in the retina (*F*_1,22_ = 1.02, *P* = 0.32), but this parameter was affected by SkQ1 (*F*_1,22_ = 16.00, *P* = 0.0007). SkQ1 supplementation decreased the* AhR* mRNA level both in OXYS rats (1.9-fold; *P* < 0.012) and in Wistar rats (1.7-fold; *P* < 0.17).

At the same time, the retinal* Cyp1a1* (Figure 1(b)),* Cyp1a2* (Figure 1(d)), and* Cyp1b1 *(Figure 1(e)) mRNA level was affected by genotype (*F*_1,26_ = 7.54, *P* = 0.011;* F*_1,24_ = 6.69, *P* = 0.016;* F*_1,25_ = 4.43, *P* = 0.047, resp.).* Post hoc* analysis showed that the retinal* Cyp1a1 *mRNA level (Figure 1(b)) was ~50% lower in OXYS rats than the Wistar strain (*P* = 0.01).* Cyp1a2* (Figure 1(d)) and* Cyp1b1 *(Figure 1(e)) mRNA expression was 2-fold (*P* = 0.03) and 1.7-fold (*P* = 0.02) higher in OXYS than in Wistar rats, respectively.

SkQ1 supplementation downregulated only* Cyp1a1 *mRNA expression (2-fold; *P* = 0.03) in the retina of Wistar rats (Figure 1(b)), whereas in the OXYS retina, the* Cyp1a1 *mRNA expression remained unchanged (Figure 1(b)). In the retina of OXYS rats, the* Cyp1a2* (Figure 1(d)) and* Cyp1b1 *(Figure 1(e)) mRNA levels were decreased 2.2-fold (*P* = 0.03) and 1.7-fold (*P* = 0.02), respectively, as a result of the dietary SkQ1 supplementation.* Cyp1a2* (Figure 1(d)) and* Cyp1b1* (Figure 1(e)) mRNA expression remained unchanged in Wistar rats between SkQ1 supplementation and control.

According to two-way ANOVA there were no statistically significant differences in the mRNA expression of* Aldh3a1* (Figure 1(c)) either between the 2 strains (*F*_1,25_ = 3.7, *P* = 0.07) or between SkQ1 supplementation and control (*F*_1,25_ = 2.5, *P* = 0.13). There were no statistically significant differences in the mRNA expression of* Aldh3a1* (Figure 1(c)) either between the 2 strains or between SkQ1 supplementation and control.

**Figure 2:**
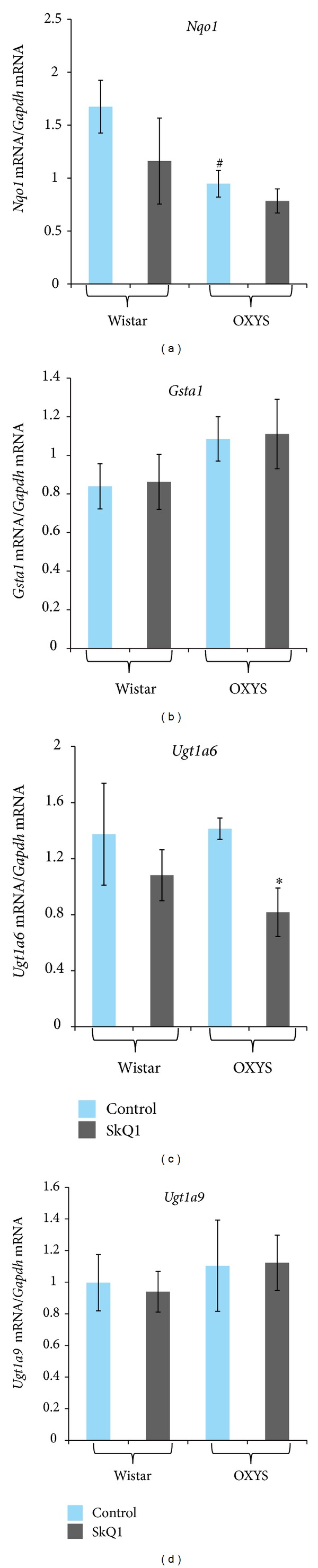
*Nrqo1* (a),* Gsta1* (b),* Ugt1a6* (c), and* Ugt1a9* (d) mRNA levels in the retinas of 3-month-old Wistar and OXYS rats treated with SkQ1. All data are normalized to the expression level of a housekeeping gene (*Gapdh*). Values are presented as mean ± SEM (*n* = 6 to 8). Significant differences between groups are marked with *P < 0.05.

Figure 2 shows mRNA levels of genes controlled by both AhR and Nrf2:* Nqo1*,* Gsta1*,* Ugt1a6*, and* Ugt1a9*. Two-way ANOVA showed that* Nqo1* mRNA level (Figure 2(a)) was affected by genotype (*F*_1,26_ = 4.48, *P* = 0.04) but was not affected by SkQ1 supplementation (*F*_1,26_ = 1.67, *P* = 0.21). One-way ANOVA showed a decreased* Nqo1* mRNA level in the retina of control Wistar rats compared to OXYS rats (1.9-fold; *P* = 0.02).

There were no statistically significant differences in the mRNA level of* Ugt1a6 *(Figure 2(c)),* Gsta1* (Figure 2(b)), and* Ugt1a9* (Figure 2(d)) between the 2 strains (*F*_1,25_ = 0.25, *P* = 0.62;* F*_1,26_ = 2.75, *P* = 0.11; and* F*_1.26_ = 0.56, *P* = 0.47, resp.) or between SkQ1 supplementation and control (*F*_1,25_ = 3.8, *P* = 0.06;* F*_1,26_ = 0.54, *P* = 0.47; and* F*_1,26_ = 0.01, *P* = 0.92, resp.).

**Figure 3:**
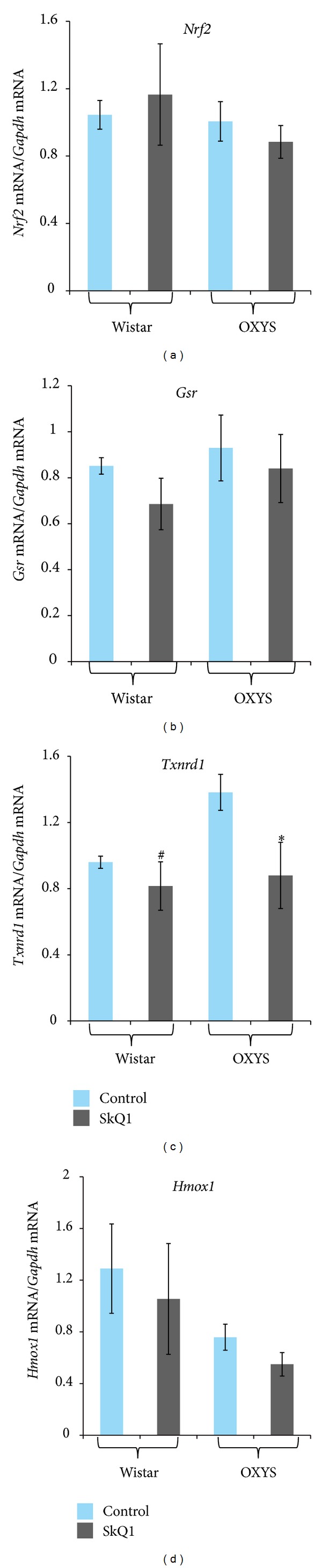
*Nrf2* (a),* Gsr* (b),* Txnrd1* (c), and* Hmox1* (d) mRNA levels in the retinas of 3-month-old Wistar and OXYS rats treated with SkQ1. All data are normalized to the expression level of a housekeeping gene (*Gapdh*). Values are presented as mean ± SEM (*n* = 6 to 8). Significant differences between groups are marked with *P < 0.05.

Figure 3 shows mRNA levels of* Nrf2* and Nrf2-dependent genes:* Gsr*,* Hmox1*, and* Txnrd1*. There were no statistically significant differences in the mRNA level of* Nrf2* (Figure 3(a)) and of Nrf2-dependent genes* Gsr* (Figure 3(b)) and* Hmox1* (Figure 3(d)) either between the 2 strains (*F*_1,26_ = 0.77, *P* = 0.39;* F*_1,26_ = 1.18, *P* = 0.29; and* F*_1,24_ = 3.57, *P* = 0.07, resp.) or between SkQ1 supplementation and control (*F*_1,26_ = 0.001, *P* = 0.997;* F*_1,26_ = 1.42, *P* = 0.24; and* F*_1,26_ = 0.17, *P* = 0.68, resp.).

Two-way ANOVA analysis showed that the mRNA level of the Nrf2-dependent gene* Txrnd1* (Figure 3(c)) was not affected by genotype (*F*_1,26_ = 1.65, *P* = 0.21) but was affected by SkQ1 supplementation (*F*_1,26_ = 5.55, *P* = 0.026). One-way ANOVA revealed a difference between the level of* Txrnd1* (Figure 3(c)) in the retina of untreated Wistar and OXYS rats (1.5-fold;* F*_1,12_ = 13.5, *P* = 0.003). SkQ1 supplementation downregulated* Txrnd1* mRNA expression (1.7-fold;* F*_1,13_ = 5.85, *P* = 0.03) in the retina of OXYS rats, whereas in the Wistar retina, the* Txrnd1* mRNA expression remained unchanged (*F*_1,13_ = 0.39, *P* = 0.54).

## 4. Discussion

The retina is among the types of tissue that are at high risk of damage by reactive oxygen species, whereas oxidative stress is a major contributor to the pathogenesis of retinopathy and AMD [22]. Our previous research showed that the mitochondria-targeted antioxidant SkQ1 can reduce clinical manifestations of retinopathy in OXYS rats. Nonetheless, the molecular mechanisms behind SkQ1's beneficial effects are not well understood. Using an animal model of retinopathy—OXYS rats—in the present work, we explored the influence of SkQ1 on mRNA expression of* AhR*,* Nrf2*, and their dependent genes; those genes can regulate oxidative and antioxidant processes in the cells.

Our present data suggest that only* AhR* and* AhR*-dependent genes of phase I of xenobiotic biotransformation were strongly sensitive to SkQ1 supplementation. The decrease of the* Ugt1a6* mRNA level and low* Nqo1 *mRNA expression in OXYS rats (compared to the Wistar strain) can be explained in part by AhR dependence of these genes. The observed effect of SkQ1 on* Txrnd1* mRNA expression was a surprise because direct links between Txrnd1 and AhR are unknown, whereas* Nrf2* and Nrf2-dependent genes* Gsr *and* Hmox1* were not affected. The absence of effects of the antioxidant SkQ1 on the genes responsible for major endogenous antioxidant systems suggests that the antioxidant activity of SkQ1 is not linked to the effects of SkQ1 on the transcription factor Nrf2 or on enzymes of phase 2 of the xenobiotic metabolism. This is not surprising; the mechanism of action of SkQ1 does not imply involvement of transcription factors or regulation of gene expression in general.

Nonetheless, we see the effects of SkQ1 on the mRNA expression of the transcription factor AhR and on mRNA levels of genes of phase I metabolism of xenobiotics that are activated by AhR. These findings are supported indirectly by the data on the influence of SkQ1 on P450 cytochrome activity in the rat liver (Grishanova et al., unpublished observations).

Differences in the level of mRNA of AhR-dependent genes are observed not only under the influence of SkQ1 but also between untreated Wistar and OXYS rats. For example, the observed level of* Cyp1a1 *mRNA is lower in OXYS rats than the Wistar strain. This situation can be explained by the significant decrease in* Cyp1a1* expression under oxidative stress [23], which constitutes one of the stages of the pathogenesis of AMD and AMD-like retinopathy in OXYS rats.

On the other hand, at baseline,* Cyp1a2 *and* Cyp1b1* mRNA levels are higher in OXYS than in Wistar rats. It is known that some P450 cytochromes can metabolize arachidonic acid to compounds that affect the tone of a vessel wall and arterial blood pressure [24–26]. In particular, Cyp1b1 participates in the synthesis of 12-HETE (12-hydroxyeicosatetraenoic acid), which is known to be cardiotoxic [27]. Arachidonic acid can be metabolized by Cyp1a2 [28]; as a result, production of epoxyeicosatrienoic acids is enhanced. The latter compounds can serve as a source of reactive oxygen species in addition to being vasodilators and proangiogenic factors able to stimulate growth of endothelial and mesangial cells [29].

Retinopathy pathogenesis includes pathological neovascularization. It has been shown that several metabolites of arachidonic acid can activate the above process. For example, cytosolic phospholipase A(2) has proangiogenic properties and stimulates pathological retinal angiogenesis [30]. Furthermore, retinal neovascularization is associated with increased 12-lipoxygenase expression and with enhanced production of 12-HETE, 15-HETE, and 5-HETE [31]. Accordingly, the elevated* Cyp1a2* and* Cyp1b1* mRNA level in OXYS rats compared to the Wistar strain is likely to reflect (at least partially) the process of pathological neovascularization in OXYS rats.

In this work, the effects of SkQ1 supplementation—where they are present—consist of downregulation of mRNA expression of the relevant genes. First, it is the reduced mRNA level of AhR both in OXYS and in Wistar rats. It is possible that the reduction in mRNA expression of AhR-controlled genes (*Cyp1a1*,* Cyp1a2*, and* Cyp1b1*) and the partially AhR-controlled gene* Ugt1a6* is mediated by SkQ1's influence on functioning of the AhR enzyme. Nonetheless, it is of course impossible to rule out direct action on the protein molecules of P450 cytochromes. Undoubtedly, detailed elucidation of such a mechanism of action of SkQ1 would be interesting, and further research is needed.

## 5. Conclusion

Our results point to the involvement of Cyp1a2 and Cyp1b1 in the pathogenesis of AMD-like retinopathy in OXYS rats. Cyp1a2 and Cyp1b1 can be considered possible therapeutic targets for novel treatments of AMD; it is plausible that these enzymes are targets of SkQ1 when it is administered systemically.

## Figures and Tables

**Table 1:** Primer sequences for analysis of gene expression.

Gene		Sequence
*Cyp1a1 *	Forward	5′-CCAAACGAGTTCCGGCCT-3′
	Reverse	5′-TGCCCAAACCAAAGAGAATGA-3′
	Probe	5′(FAM)-TTCTCACTCAGGTGTTTGTCCAGAGTGCC-(BHQ1)3′

*Cyp1a2 *	Forward	5′-CGCCCAGAGCGGTTTCTTA-3′
	Reverse	5′-TCCCAAGCCGAAGAGCATC-3′
	Probe	5′(FAM)-CAATGACAACACGGCCATCGACAAG-(BHQ1)3′

*Cyp1b1 *	Forward	5′-GGCATCGCACTTGTACTTCG-3′
	Reverse	5′-CACCAGAGCCTGATGGATGG-3′
	Probe	5′(FAM)-TCTCGCCATTCAGCACCACCACGG-(BHQ1)3′

*Gsta1 *	Forward	5′-ACTACATTGCCACCAAATACAACCT-3′
	Reverse	5′-CACTCCTTCTGCATACATGTCGAT-3′
	Probe	5′(FAM)-ATGGGAAGGACATGAAGGAGAGAGCCC-(BHQ1)3′

*Nqo1 *	Forward	5′-TTGAGTCATCTCTGGCGTATAAGG-3′
	Reverse	5′-GGTCTGCAGCTTCCAGCTTT-3′
	Probe	5′(FAM)-AGGCCGCCTGAGCCCGGATA-(BHQ1)3′

*Aldh3a1 *	Forward	5′-CCGTGATTATGGGAGGATCATC-3′
	Reverse	5′-TGGGCTACTTTCTGGTTGTCAAT-3′
	Probe	5′(FAM)-TGACCGTCACTTCCAGCGGGTCA-(BHQ1)3′

*Ugt1a6 *	Forward	5′-CCTTGGACGTGATTGGCTTT-3′
	Reverse	5′-GCAGCCATAGGCACAACTTTTATA-3′
	Probe	5′(FAM)-CTGGCCATCGTGTTGACGGTGGT-(BHQ1)3′

*Ugt1a9 *	Forward	5′-GAGGCTTTGGGCAGAATTCC-3′
	Reverse	5′-TTTGCAAGGTTCGATGGTCTAGTT-3′
	Probe	5′(FAM)-CAGACGGTCCTGTGGCGCTACACC-(BHQ1)3′

*AhR *	Forward	5′-TGGACAAACTCTCCGTTCTAAGG-3′
	Reverse	5′-GATTTTAATGCAACATCAAAGAAGCT-3′
	Probe	5′(FAM)-CAGCGTCACGTACCTGAGGGCCA-(BHQ1)3′

*Nrf2 *	Forward	5′-AGCAACTCCAGAAGGAACAGGAGA-3′
	Reverse	5′-CTTGTTTGGGAATGTGGGCAACCT-3′
	Probe	5′(FAM)-TCCCAATTCAGCCAGCCCAGCACA-(BHQ1)3′

*Hmox1 *	Forward	5′-TTACACACCAGCCACACAGCACTA-3′
	Reverse	5′-CATGGCCTTCTGCGCAATCTTCTT-3′
	Probe	5′(FAM)-FAMTGAGCTGCTGGTGGCCCACGCATATA-(BHQ1)3′

*Txnrd1 *	Forward	5′-TTTACTCAGCAGAGCGGTTCCT-3′
	Reverse	5′-TGCACATTCCAAGGCGACAT-3′
	Probe	5′(FAM)-AAGACCCTAGTGGTTGGCGCGTCCT-(BHQ1)3′

*Gsr *	Forward	5′-CTTCGACAATACGGTCGCCATTCA-3′
	Reverse	5′-AATCTATAAAGCTGGCGCAGGACG-3′
	Probe	5′(FAM)-AGTGGGCCTCTGGGAGGAACCAATCA-(BHQ1)3′

*Gapdh *	Forward	5′-CAAGGTCATCCATGACAACTTTG-3′
	Reverse	5′-GGGCCATCCACAGTCTTCTG-3′
	Probe	5′(FAM)-ACCACAGTCCATGCCATCACTGCCA-(BHQ1)3′

## Abbreviations

AhR: Aryl hydrocarbon receptor
Aldh3a1: Aldehyde dehydrogenase 3A1
AMD: Age-related macular degeneration
ARE: Antioxidant-responsive elements
Arnt: Ah receptor nuclear translocator
BHQ: Black hole quencher
Cyp1a1: Cytochrome P450 1A1
Cyp1a2: Cytochrome P450 1A2
Cyp1b1: Cytochrome P450 1B1
FAM: 6-Carboxyfluorescein
Gsr: Glutathione-S-reductase
Gsta1: Glutathione S-transferase A1
Hmox1: Heme oxygenase 1
Nqo1: NADPH-quinone oxidoreductase
Nrf2: Nuclear factor, erythroid derived 2
Txnrd1: Thioredoxin reductase 1
Ugt1a6: UDP-glucuronosyltransferase 1A6
Ugt1a9: UDP-glucuronosyltransferase 1A9
VEGF: Vascular endothelium growth factor
XRE: Xenobiotic responsive element.
